# Large‐scale transcriptome profiles reveal robust 20‐signatures metabolic prediction models and novel role of G6PC in clear cell renal cell carcinoma

**DOI:** 10.1111/jcmm.15536

**Published:** 2020-06-21

**Authors:** Wen‐Hao Xu, Yue Xu, Xi Tian, Aihetaimujiang Anwaier, Wang‐Rui Liu, Jun Wang, Wen‐Kai Zhu, Da‐Long Cao, Hong‐Kai Wang, Guo‐Hai Shi, Yuan‐Yuan Qu, Hai‐Liang Zhang, Ding‐Wei Ye

**Affiliations:** ^1^ Department of Urology Fudan University Shanghai Cancer Center Shanghai China; ^2^ Department of Oncology Shanghai Medical College Fudan University Shanghai China; ^3^ Department of Ophthalmology First Affiliated Hospital of Soochow University Suzhou China; ^4^ Medical College Soochow University Suzhou China; ^5^ Department of Neurosurgery Affiliated Hospital of Youjiang Medical College for Nationalities Guangxi China

**Keywords:** clear cell renal cell carcinoma, G6PC, immune infiltration, metabolic prediction models, prognosis, tumour microenvironment

## Abstract

Clear cell renal cell carcinoma (ccRCC) is the most common and highly malignant pathological type of kidney cancer. We sought to establish a metabolic signature to improve post‐operative risk stratification and identify novel targets in the prediction models for ccRCC patients. A total of 58 metabolic differential expressed genes (MDEGs) were identified with significant prognostic value. LASSO regression analysis constructed 20‐mRNA signatures models, metabolic prediction models (MPMs), in ccRCC patients from two cohorts. Risk score of MPMs significantly predicts prognosis for ccRCC patients in TCGA (*P *< 0.001, HR = 3.131, AUC = 0.768) and CPTAC cohorts (*P *= 0.046, HR = 2.893, AUC = 0.777). In addition, *G6PC*, a hub gene in PPI network of MPMs, shows significantly prognostic value in 718 ccRCC patients from multiply cohorts. Next, G6Pase was detected high expressed in normal kidney tissues than ccRCC tissues. It suggested that low G6Pase expression significantly correlated with poor prognosis (*P *< 0.0001, HR = 0.316) and aggressive progression (*P *< 0.0001, HR = 0.414) in 322 ccRCC patients from FUSCC cohort. Meanwhile, promoter methylation level of *G6PC* was significantly higher in ccRCC samples with aggressive progression status. *G6PC* significantly participates in abnormal immune infiltration of ccRCC microenvironment, showing significantly negative association with check‐point immune signatures, dendritic cells, Th1 cells, etc. In conclusion, this study first provided the opportunity to comprehensively elucidate the prognostic MDEGs landscape, established novel prognostic model MPMs using large‐scale ccRCC transcriptome data and identified G6PC as potential prognostic target in 1,040 ccRCC patients from multiply cohorts. These finding could assist in managing risk assessment and shed valuable insights into treatment strategies of ccRCC.

## 1 INTRODUCTION

Renal cell carcinoma is one of the most common malignant tumours of the urogenital system, accounting for about 5% of all new cases of adult males and 3% of new cases of females.^1^ According to statistics in the United States, there are about 73 820 new cases of kidney cancer and 14 770 deaths in 2019.^2^ Clear cell renal cell carcinoma (ccRCC) is the most common and highly malignant pathological type of kidney cancer (accounting for 70%‐85%). About 25%‐30% of ccRCC patients have metastases at first diagnosis, and the 5‐year survival rate of metastatic ccRCC is only 32%. In addition, even ccRCC patients who are initially effective in treatment will have disease progression after a period of time, at which time most patients will lack subsequent effective treatment.

In recent years, new immunotherapy represented by PD‐1/PD‐L1, CTLA4 inhibitors has rapidly emerged in the field of ccRCC treatment and has shown encouraging results for patients with advanced refractory disease.^3^ In 2020, ASCO GU published the 5‐year follow‐up results of the CheckMate 025 study, showing the 5‐year survival rate of monoclonal antibody second‐line treatment is as high as 26%, which demonstrates the advantages of immunotherapy's survival benefits and new chapter of treatment strategies for high‐risk ccRCC patients.^4^, ^5^, ^6^ Immune check‐point inhibitors combined with TKI play a variety of roles from the perspective of inducing anti‐tumour immune normalization, inhibiting the development of advanced ccRCC and regulating tumour microenvironment (TME). Its success largely depends on deep understanding of tumour cells and TME interaction.^7^, ^8^ With the deepening of research, more evidence shows that not only the efficacy of immunotherapy depends on the activation of the tumour immune microenvironment, but also the efficacy of traditional treatments such as targeted therapy also depends on the strength of individual anti‐tumour immune response.^9^, ^10^, ^11^ Thus, it is of great significance exploring the underlying mechanism of TME‐driven tumorigenesis and development, improving the efficiency of various existing treatments and discovering novel precise targets for ccRCC therapies.

In TME, tumour cells and immune cells reprogram their metabolic patterns to adapt to the microenvironment of hypoxia, acidity and low nutrition.^12^ For example, tumour cells show enhanced aerobic glycolysis (Warburg effect) but reduce oxidative phosphorylation, which has a great effect on T cell‐mediated anti‐tumour immune response and tumour‐infiltrating myeloid cell activity; macrophages tend to be M2‐type polarization, showing up‐regulated fatty acid synthesis and β‐oxidation.^13^ The activation of tumour cell pro‐cancer signals not only affects its own malignant biological behaviour, but also promotes the development of tumours.^14^ It can also deflect the functional phenotype of tumour‐infiltrating immune cells by changing the metabolic secretion profile and TME of tumour cells and induce the formation of tumour immune escape.^14^, ^15^ Therefore, the metabolic reprogramming of tumour cells and immune cells is crucial for understanding the game process of tumour cells' evil behaviour, tumour immune response and tumour immune escape and provides new directions for regulating tumour immunity.

The methods of constructing clinical prediction models based on retrospective data help researches realize the prediction of clinical outcomes with several prognostic factors, which is more likely to change our clinical practice and has strong clinical guidance value.^16^ In 2018, RCClnc4 has been demonstrated to have precise prognostic significance in early ccRCC. This study aimed to first establish and validate an effective prognostic metabolic prediction models enrolled large‐scale transcriptome metabolic genes for ccRCC patients. We suggested that the metabolic prediction models (MPMs) classifier could facilitate risk management and treatment strategies for ccRCC patients and identify novel targets in the MPMs co‐network.

## 2 MATERIALS AND METHODS

### 2.1 Raw data collection and processing

Publicly available mRNA expression and clinical data from ccRCC cohorts were used in this study. Consents and ethical approval of enrolled patients are available in the related original articles where the data sets were published. A total of 718 ccRCC patients from online data sets, including 534 ccRCC and 72 normal samples obtained from The Cancer Genome Atlas (TCGA) database (https://portal.gdc.cancer.gov/), 93 ccRCC and 20 normal samples obtained from Clinical Proteomic Tumor Analysis Consortium (CPTAC) and 91 ccRCC samples obtained from RECA‐EU (available in International Cancer Genome Consortium, ICGC), were included in this study.

### 2.2 Identification of metabolic differentially expressed genes

Forty‐one metabolic pathways were selected according to KEGG pathways atlas. The 911 metabolic genes were utilized for identification of significant metabolic differentially expressed genes (MDEGs) using *Limma* R package (Version 3.6.3) with FDR < 0.05 and |logFC|>0.5. The intersective metabolic genes between TCGA and CPTAC cohorts were selected for further analyses.

### 2.3 Development of metabolic prediction models (MPMs) and survival analysis

Univariate Cox regression analysis was used to identify prognostic implications of significant MDEGs and presented in forest plot using the *survival* R package. Then, LASSO regression analysis was performed to construct the 20‐mRNA signatures model, metabolic prediction models (MPMs), in ccRCC patients from TCGA and CPTAC cohorts with the *glmnet* and *survival* R package. A risk score of each ccRCC patient was calculated on the basis of MPMs, and ccRCC patients were thus divided into low‐ and high‐risk groups.

For survival analyses, we selected TCGA and CPTAC cohorts with relevant long‐term survival data from patients at the time of surgical resection and pathologically diagnosed as ccRCC. Survival data were of two types: overall survival and progression‐free survival. Log‐rank test in separate curves and Kaplan‐Meier method with 95% confidence intervals (95%CI) were utilized to performing the follow‐up duration analysis. Meanwhile, survival risk assessment of MPMs and hierarchical clustering was shown in patients from TCGA or CPTAC cohort.

### 2.4 Cox regression analysis and receiver operating characteristic curve construction

All ccRCC patients from TCGA and CPTAC cohorts with complete transcriptome information and relevant clinical pathologic parameters were included for subsequent analysis. Univariate and multivariable Cox regression analyses were used to evaluate the independent prognostic value of the metabolic clusters using forest plot with the *survival* R package. The receiver operating characteristic curve (ROC) was constructed for traditional clinical pathologic parameter and the risk score of MPMs in both TCGA and CPTAC cohorts using *survival ROC* R package. The area under the curve (AUC) was utilized to assess the predictive value of these prognostic signatures. In addition, Nomogram was developed on the basis of all the independent prognostic factors in TCGA cohort.

### 2.5 Gene set enrichment analyses

Gene set enrichment analyses (GSEA) were performed with a permutation test with 1000 times to find the top enriched signal pathways and significantly involved metabolic pathways the Molecular Signatures Database v4.0 (MSigDB) with Adj. *P *< 0.01 and FDR < 0.25.

### 2.6 Tumour microenvironment purity assessment

ESTIMATE algorithm was utilized to evaluate total and immune scores using *estimate* package (http://r‐forge.rproject.org; repos = rforge, dependencies = TRUE) in patients from TCGA cohort. Associated between tumour microenvironment (TME) purity and risk score of MPMs or G6PC expression was assessed using Pearson's r test.

### 2.7 Differential G6PC mRNA expression and survival analysis

Protein‐protein interaction network of 20 signatures in MPMs was constructed using Search Tool for the Retrieval of Interacting Genes (STRING; http://string‐db.org, version 10.0) online database. Differential expressed *G6PC* level was evaluated between ccRCC and normal samples from TCGA, CPTAC and RECA‐EU cohort using Student's t test. Survival analysis of G6PC predicting prognosis ability was performed with GEPIA (http://gepia.cancer‐pku.cn/detail.php###) in patients from TCGA cohort with cut‐off value set as median. Kaplan‐Meier method with 95% confidence intervals (95% CI) and log‐rank test were used in survival analysis in CPTAC, RECA‐EU and FUSCC. Best cut‐off values were set using X‐tile software. All patients at risk or patients numbers in different risk groups were shown in all survival plots.

### 2.8 Glucose‐6‐phosphatase (G6Pase) expression in ccRCC and normal samples

G6Pase protein expression, coded by G6PC gene, was detected in ccRCC and normal samples from the human protein atlas (https://www.proteinatlas.org/) and immunohistochemistry (IHC) data, including staining quantity, intensity, location and patients’ data, were available online. Formalin‐fixed, paraffin‐embedded ccRCC tissues and human renal tissues were stained for anti‐G6Pase using ab243319 (Abcam, USA) at 1/3000 dilution in FUSCC cohort and then independently evaluated by two experienced pathologists. The overall IHC score ranging from 0 to 12 was measured based on the multiply of the staining intensity and extent score, as previously described.^17^ Low G6Pase expression group scores from 0 to 2, and high G6Pase group scores from 3 to 12.

### 2.9 Immune cell infiltrations of G6PC in ccRCC

TIMER (Tumor IMmune Estimation Resource, https://cistrome.shinyapps.io/timer/) is a web server for evaluating systematic various immune cells infiltration and clinical implications. In this study, correlation between infiltration of immune cells and G6PC copy number variation and expression levels were performed.

### 2.10 Statistical analysis

All analyses were performed in the R (Version 3.6.0) and RStudio (Version 1.2.1335) and GraphPad Prism 7. Unless otherwise stated, results were considered statistically significant when *P*‐value < 0.05. Two‐sided and *p*‐values less than 0.05 were taken as significant in all tests.

## 3 RESULTS

### 3.1 Identification of metabolic differential expressed genes (MDEGs) in both TCGA and CPTAC cohorts

**FIGURE 1:**
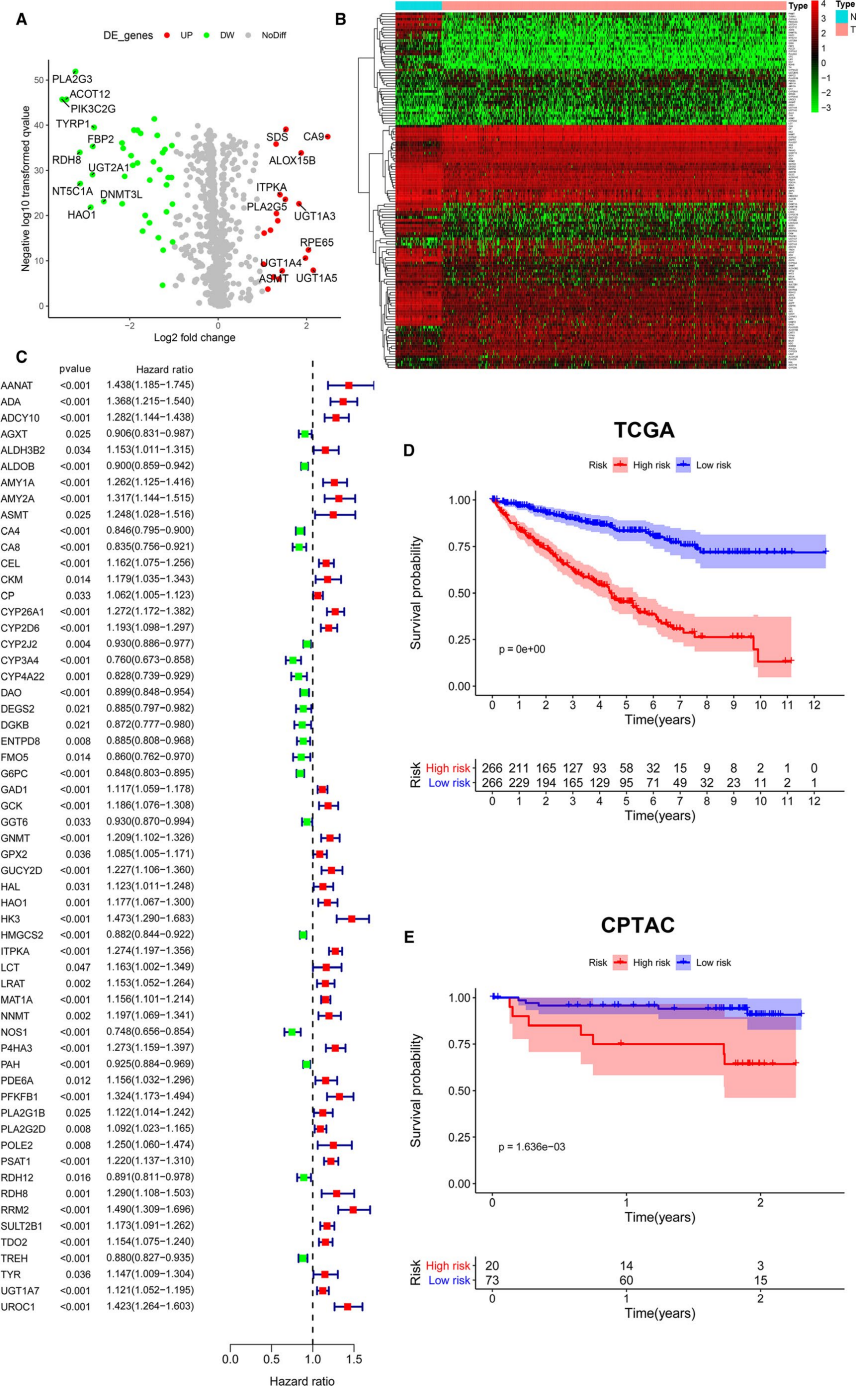
Identification of MDEGs in both TCGA and CPTAC cohorts. A, Identification of significant MDEGs using Limma package in 905 metabolic genes. B, Hierarchical partitioning of significant MDEGs was acquired from DNA microarrays based on TCGA cohort. The mRNA expression of these genes was performed across 534 ccRCC patients and 72 normal people with high in red and low in green. C, Univariate Cox regression analysis of 58 significant MDEGs (*P *< 0.05) in TCGA cohort was performed in a forest plot. D, The 20‐mRNA signatures model (MPMs) in ccRCC patient was calculated using LASSO regression analysis. Kaplan‐Meier survival analysis showed significant predictive value of the risk score depending on MPMs in TCGA cohort. E, Kaplan‐Meier survival analysis showed significant predictive value of the risk score depending on MPMs in CPTAC cohort. High‐risk group was marked in red, and low‐risk group was marked in blue. MDEGs, metabolic differential expressed genes; MPMs, metabolic prediction models; ccRCC, clear cell renal cell carcinoma; TCGA, the cancer genome atlas; CPTAC, Clinical Proteomic Tumor Analysis Consortium

The expression of 911 metabolic genes was collected from 534 ccRCC and 72 normal samples in TCGA cohort. At the same time, 905 of 911 metabolic genes were also found from 56 ccRCC patients and 47 normal people in CPTAC cohort. Then, these 905 metabolic genes were utilized for further analysis. 133 significant MDEGs were identified in 905 metabolic genes and visualized in volcano plot (Figure 1A). Hierarchical partitioning of significant MDEGs was acquired from DNA microarrays based on TCGA cohort (Figure 1B). The mRNA expression of these genes was performed across 534 ccRCC patients and 72 normal people with high in red and low in green. Meanwhile, univariate Cox regression analysis of 58 significant MDEGs (*P *< 0.05) in TCGA cohort was performed in a forest plot (Figure 1C). Markedly, LASSO regression analysis constructed 20‐mRNA signatures model, metabolic prediction models (MPMs), in ccRCC patients of TCGA or CPTAC cohort. Kaplan‐Meier survival analysis showed significant predictive value of the risk score depending on MPMs in TCGA (Figure 1D) or CPTAC cohort (Figure 1E). High‐risk group was marked in red, and low‐risk group was marked in blue.

### 3.2 Survival risk assessment of MPMs in TCGA or CPTAC cohort

**FIGURE 2:**
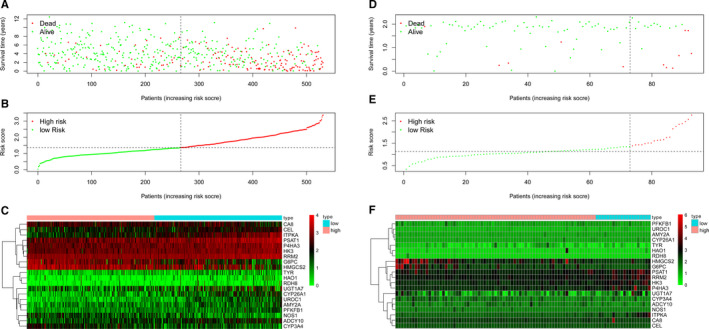
Survival risk assessment of MPMs consists of metabolic 20‐mRNA signatures in TCGA and CPTAC cohorts. A‐C, The distribution of survival time, status, risk score and hierarchical partitioning of 20 signatures in tumour and normal samples was shown in TCGA cohort. D‐F, The distribution of survival time, status, risk score and hierarchical partitioning of 20 signatures in tumour and normal samples was shown in CPTAC cohort. MPMs, metabolic prediction models; TCGA, the cancer genome atlas; CPTAC, Clinical Proteomic Tumor Analysis Consortium

Survival risk assessment of MPMs consisting of metabolic 20‐mRNA signatures was performed in TCGA or CPTAC cohort. The distribution of survival time, status (Figure 2A), risk score (Figure 2B) and hierarchical partitioning (Figure 2C) of MPMs in tumour and normal samples was shown in TCGA cohort. Meanwhile, the distribution of survival time, status (Figure 2D), risk score (Figure 2E) and hierarchical partitioning (Figure 2F) of MPMs in tumour and normal samples was shown in CPTAC cohort.

### 3.3 Cox regression analysis, ROC analysis and Nomogram of independent prognostic factors and MPMs in ccRCC patients

**FIGURE 3:**
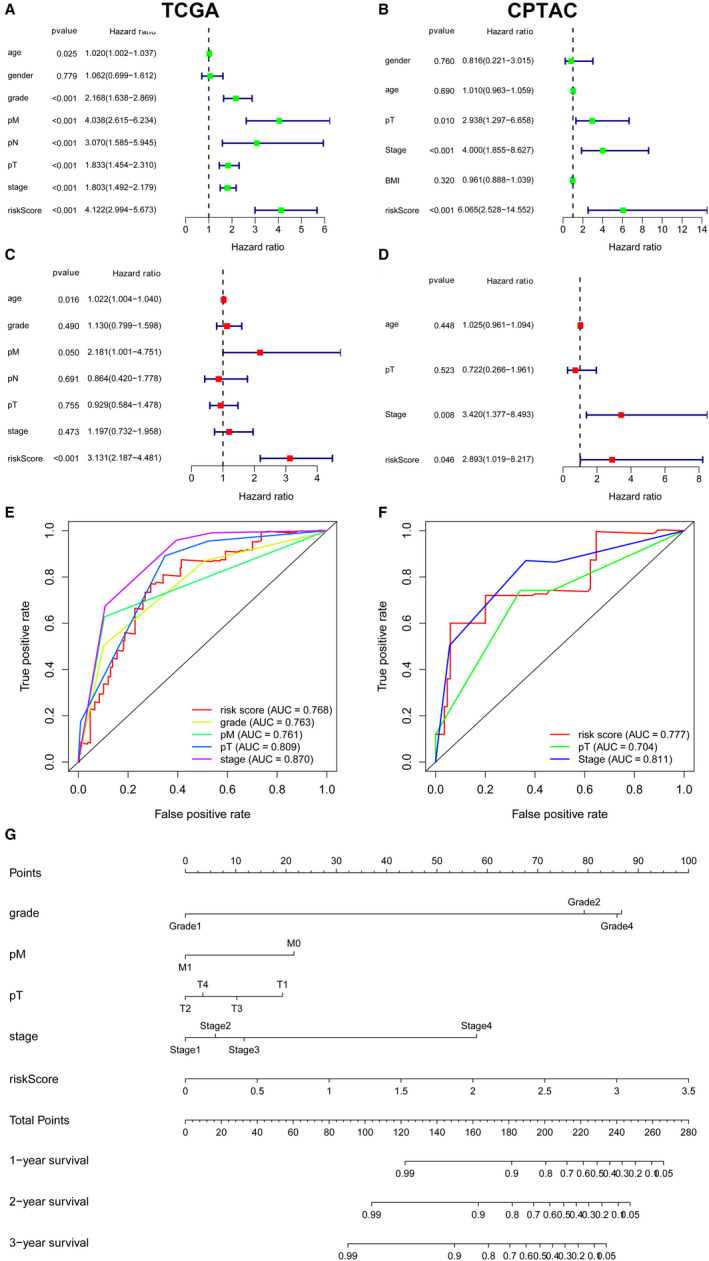
Cox regression analysis, ROC analysis and Nomogram of independent prognostic factors and MPMs in ccRCC patients. A‐D, Univariate and multivariate Cox regression analysis enrolling clinical pathologic parameters and MPMs were illustrated in TCGA and CPTAC cohorts using forest plots. Risk score of MPMs significantly predicts prognosis for ccRCC patients in TCGA (*P *< 0.001, HR = 3.131) and CPTAC cohorts (*P *= 0.046, HR = 2.893). E‐F, ROC analysis showed robust predictive value of MPMs in TCGA (AUC = 0.768) and CPTAC (AUC = 0.777) cohorts. G, A Nomogram was constructed based on 5 independent prognostic factors, including ISUP grade, pathologic M stage, pathologic T stage, AJCC stage and risk score of MPMs, in ccRCC patients. MPMs, metabolic prediction models; ccRCC, clear cell renal cell carcinoma; TCGA, the cancer genome atlas; CPTAC, Clinical Proteomic Tumor Analysis Consortium

Univariate and multivariate Cox regression analysis enrolling clinical pathologic parameters and MPMs were illustrated in TCGA and CPTAC cohorts using forest plots (Figure 3A‐D). Risk score of MPMs significantly predicts prognosis for ccRCC patients in TCGA (*P *< 0.001, HR = 3.131) and CPTAC cohorts (*P *= 0.046, HR = 2.893). In addition, ROC analysis showed robust predictive value of MPMs in TCGA (AUC = 0.768) and CPTAC (AUC = 0.777) cohorts (Figure 3E‐F). A Nomogram was constructed based on 5 independent prognostic factors, including ISUP grade, pathologic M stage, pathologic T stage, AJCC stage and risk score of MPMs, in ccRCC patients (Figure 3G).

### 3.4 KEGG pathways analysis using GSEA

**FIGURE 4:**
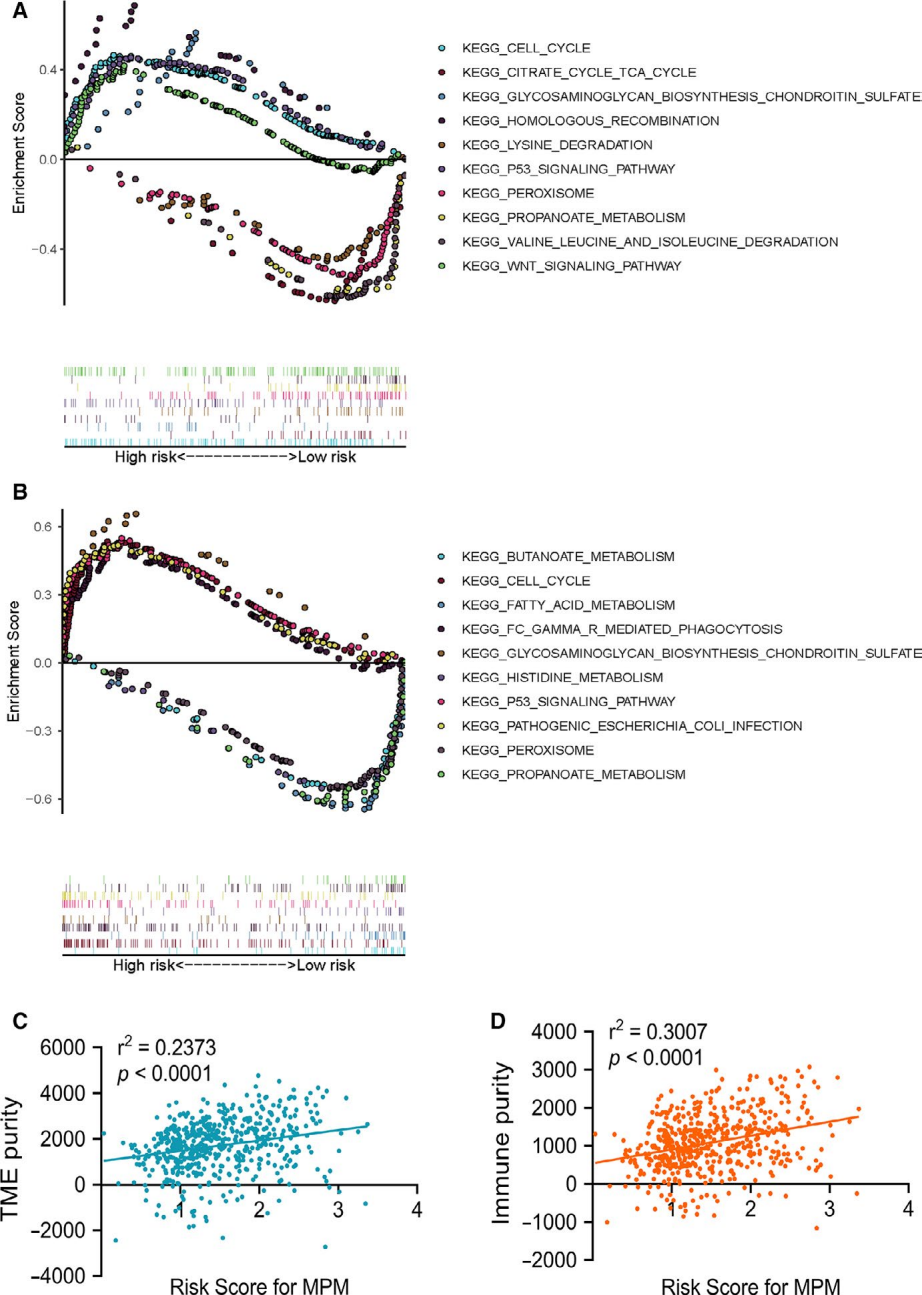
GSEA indicated significantly altered KEGG pathways based on differential risk score of MPMs in ccRCC patients with available transcriptomics data from TCGA and CPTAC cohorts. A, Top 5 significantly altered KEGG pathways in high‐ or low‐risk ccRCC patients in TCGA cohort. B, Top 5 significantly altered significant KEGG pathways in high‐ or low‐risk ccRCC patients of CPTAC cohort. C, Tumour environment purity was measured using ESTIMATE algorithm and showed a significant relationship with risk score of MPMs in ccRCC patients from TCGA cohort (*r* ^2^ = 0.2373, *P *< 0.0001). D, Immune purity in ccRCC environment significantly correlated with risk score of MPMs (*r* ^2^ = 0.3007, *P *< 0.0001). GSEA, gene set enrichment analysis; KEGG, Kyoto Encyclopedia of Genes and Genomes; MPMs, metabolic prediction models; ccRCC, clear cell renal cell carcinoma; TCGA, the cancer genome atlas; CPTAC, Clinical Proteomic Tumor Analysis Consortium

GSEA indicated significantly altered KEGG pathways based on differential risk score of MPMs in ccRCC patients with available transcriptomics data from TCGA and CPTAC cohorts. Top 5 significantly altered KEGG pathways in high‐ or low‐risk ccRCC patients were performed in TCGA (Figure 4A) or CPTAC (Figure 4B) cohort. Metabolic significant KEGG pathways in high‐ or low‐risk ccRCC patients were performed in TCGA (Figure S1A) or CPTAC (Figure S1B) cohort. Tumour environment purity was measured using ESTIMATE algorithm, which showed a significant relationship with risk score of MPMs in ccRCC patients from TCGA cohort (*r* ^2^ = 0.2373, *P *< 0.0001) (Figure 4C). Meanwhile, immune purity in ccRCC environment significantly correlated with risk score of MPMs (*r* ^2^ = 0.3007, *P *< 0.0001) (Figure 4D).

### 3.5 The hub gene in PPI network of MPMs

**FIGURE 5:**
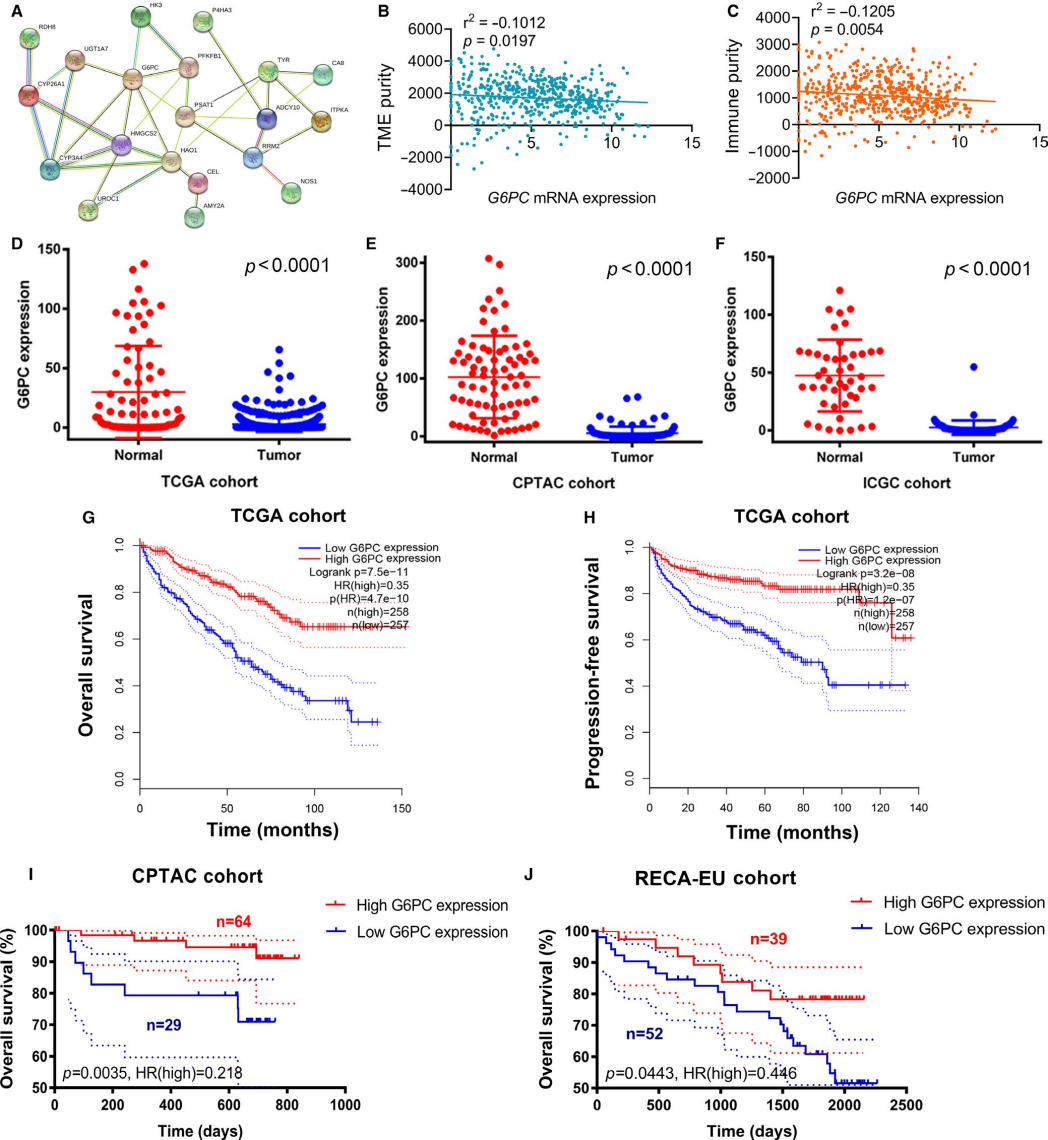
*G6PC*, a hub gene in PPI network of MPMs, shows significant prognostic value in 699 ccRCC patients from TCGA, CPTAC and RECA‐EU cohorts. A, PPI network was constructed in 20 metabolic mRNA signatures in MPMs. B and C, *G6PC* mRNA expression showed a negatively relationship with tumour environment purity (*r* ^2^ = −0.1012, *P *< 0.0001) and immune purity (*r* ^2^ = −0.1205, *P *< 0.0001) in ccRCC. D‐F, Differential mRNA expression of *G6PC* in ccRCC and adjacent normal tissues was displayed based on TCGA, CPTAC and RECA‐EU (public data at ICGC) cohorts. G‐H, Kaplan‐Meier survival analysis indicated that low *G6PC* mRNA expression level significantly correlated with poor OS (*P *< 0.0001, HR = 0.35) and PFS (*P *< 0.0001, HR = 0.35). I, Low *G6PC* mRNA expression level was significantly associated with poor prognosis in CPTAC (*P *= 0.0035, HR = 0.218) and ICGA (*P *= 0.0443, HR = 0.446) cohorts. PPI, protein‐protein interaction; MPMs, metabolic prediction models; ccRCC, clear cell renal cell carcinoma; TCGA, the cancer genome atlas; CPTAC, Clinical Proteomic Tumor Analysis Consortium; ICGC, International Cancer Genome Consortium

*G6PC*, a hub gene in PPI network of MPMs, shows significant prognostic value in 699 ccRCC patients from TCGA, CPTAC and ICGC cohorts. PPI network was constructed in 20 metabolic mRNA signatures in MPMs (Figure 5A). Interestingly, because of relatively low protein expression in tumour samples, the mRNA and proteome expression levels are in significant linear relationship (Figure S2A, *P *< 0.001, *r* = 0.371). In transcriptional levels, transcription factor regulation, related LncRNA, targeted miRNA, activation and inhibition of *G6PC* networks were constructed in Figure S2B *G6PC* mRNA expression showed a negatively relationship with tumour environment purity (*r* ^2^ = −0.1012, *P *< 0.0001) and immune purity (*r* ^2^ = −0.1205, *P *< 0.0001) in ccRCC (Figure 5B and C). Differential mRNA expression of *G6PC* in ccRCC and adjacent normal tissues was displayed based on TCGA, CPTAC and RECA‐EU cohorts (Figure 5D‐F). In addition, Kaplan‐Meier survival analysis indicated that low *G6PC* mRNA expression level significantly correlated with poor OS (*P *< 0.0001, HR = 0.35) and PFS (*P *< 0.0001, HR = 0.35) in TCGA cohort (Figure 5G and H). Low *G6PC* mRNA expression level was significantly associated with poor prognosis in CPTAC (*P *= 0.0035, HR = 0.218) and RECA‐EU (*P *= 0.0443, HR = 0.446) cohorts (Figure 5I and J).

### 3.6 Differential G6Pase expression predicts outcomes in FUSCC cohort

**FIGURE 6:**
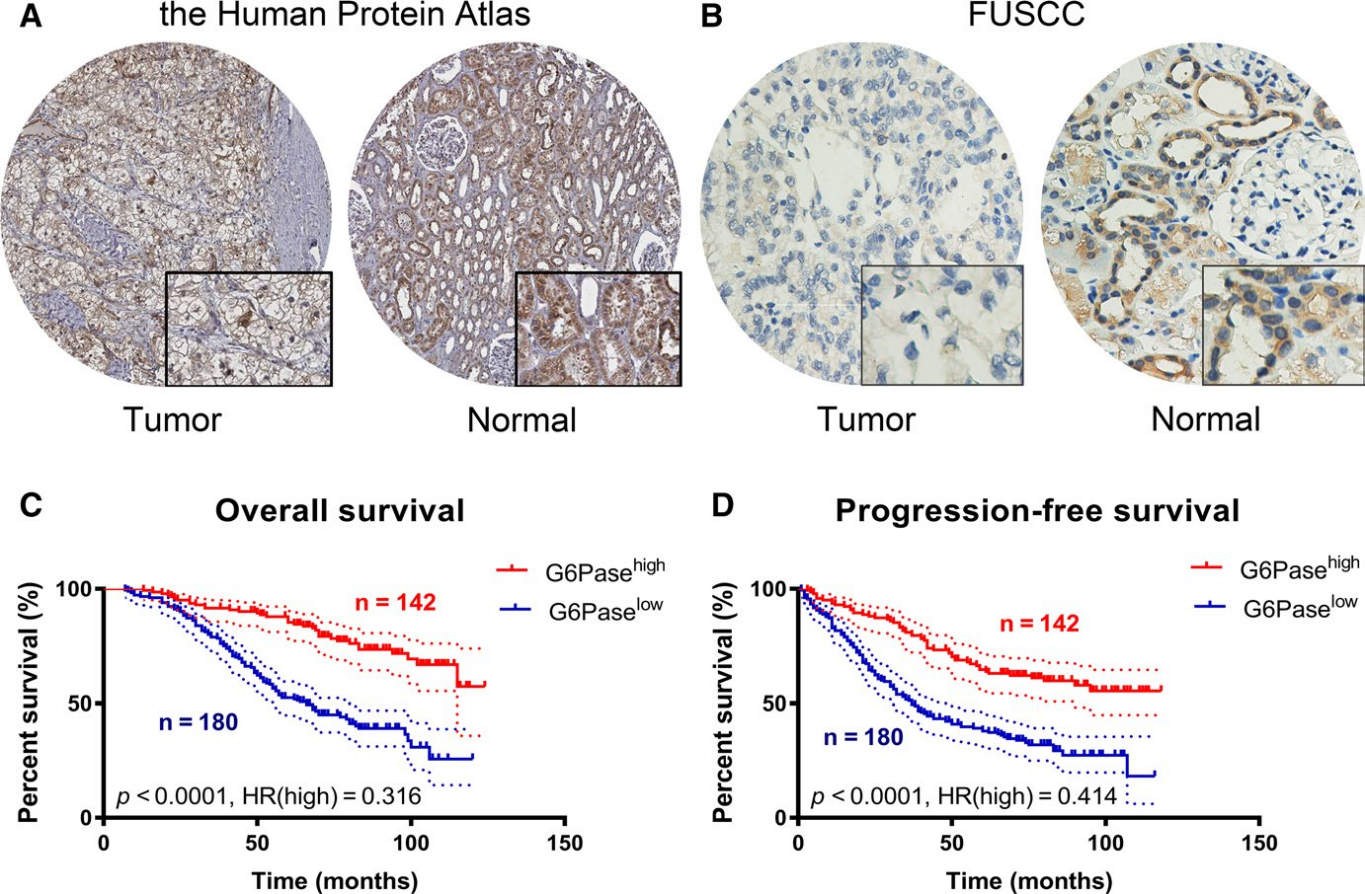
Differential G6Pase expression predicts outcomes in 322 ccRCC patients from FUSCC cohorts. A, G6Pase was detected high expressed in normal kidney tissues (specifically in tubules cells rather than glomeruli cells), while not detected in ccRCC tissues from the Human Protein atlas. B, Significantly elevated G6Pase expression was found in normal tissues compared with ccRCC tissues from FUSCC cohort. C‐D, Low G6Pase expression was significantly correlated with poor prognosis (*P *< 0.0001, HR = 0.316) and aggressive progression (*P *< 0.0001, HR = 0.414) in 322 ccRCC patients from FUSCC cohort. ccRCC, clear cell renal cell carcinoma; FUSCC, Fudan University Shanghai Cancer Center

A total of 322 ccRCC patients from FUSCC cohort were enrolled. G6Pase was detected high expressed in normal kidney tissues (specifically in tubules cells rather than glomeruli cells), while not detected in ccRCC tissues from the Human Protein atlas (Figure 6A). Meanwhile, significantly elevated G6Pase expression was found in normal tissues compared with ccRCC tissues from FUSCC cohort (Figure 6B). Clinicopathological characteristics in relation to G6Pase expression status were shown in 322 ccRCC patients from FUSCC cohort (Table S1). Traditionally clinicopathological factors, such as TNM stage or ISUP grade, were significantly correlated with G6Pase expression level in tumour samples (*P *< 0.05). In addition, low G6Pase expression was significantly correlated with poor prognosis (*P *< 0.0001, HR = 0.316) and aggressive progression (*P *< 0.0001, HR = 0.414) in 322 ccRCC patients from FUSCC cohort (Figure 6C and D).

### 3.7 Most co‐expressed genes and promoter methylation levels of G6PC in ccRCC

**FIGURE 7:**
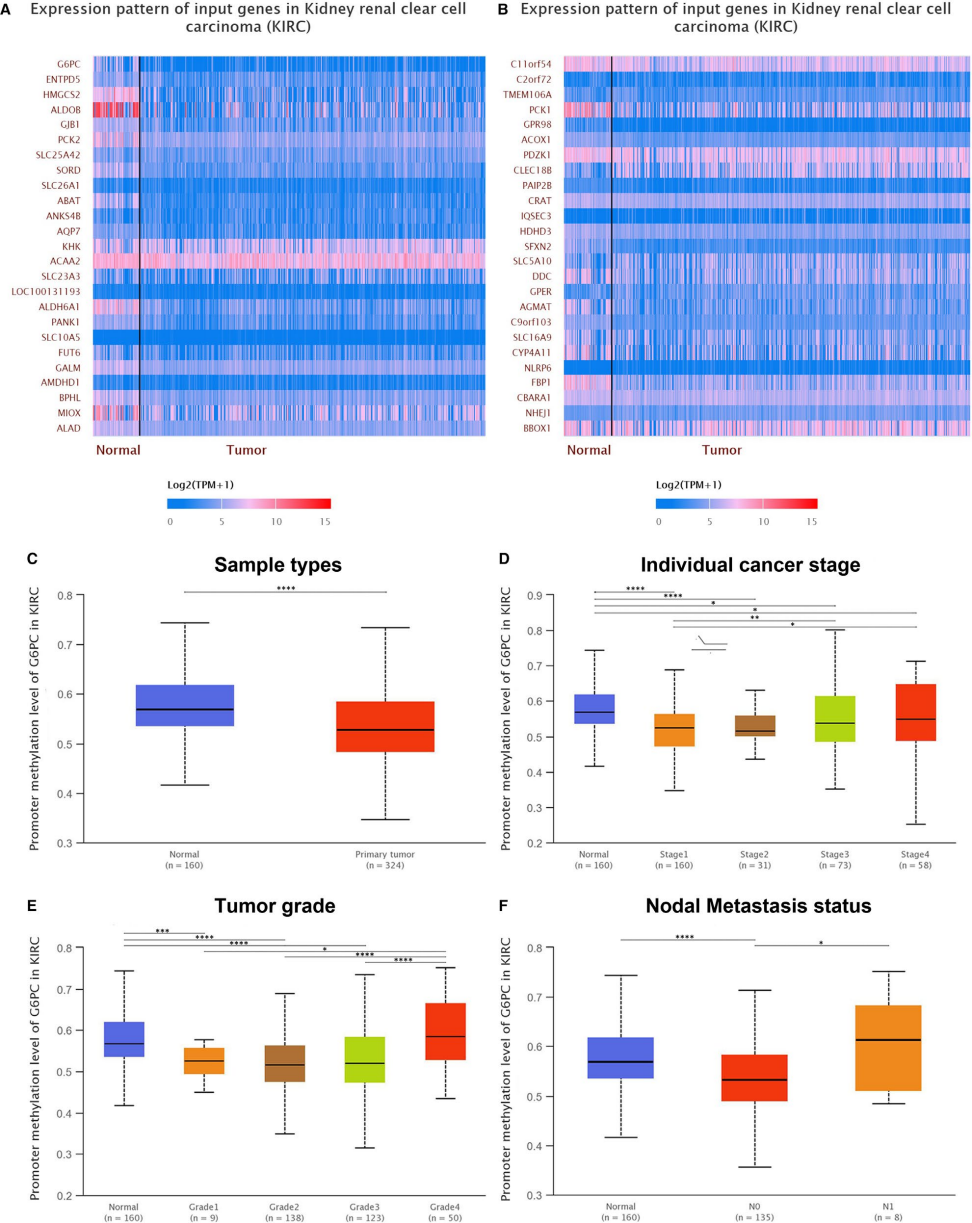
Most co‐expressed genes and promoter methylation levels of *G6PC* in ccRCC. A and B, Top 50 co‐expression genes with *G6PC* were extracted and shown in heat map in ccRCC. C, Promoter methylation levels of *G6PC* were significantly lower in primary ccRCC samples than normal samples (*P *< 0.0001). D, Promoter methylation levels of *G6PC* significantly climbed with elevated individual cancer stage and were the highest in samples with stage 4. E, Promoter methylation levels of *G6PC* significantly climbed with elevated individual tumour grade and were the highest in samples with grade 4. F, Promoter methylation levels of *G6PC* were significantly higher in ccRCC samples with nodal metastasis compared with pN0 patients (*P *< 0.05). ccRCC, clear cell renal cell carcinoma; pN0, pathological negative nodal metastasis status

Top 50 co‐expression genes with *G6PC* were extracted and shown in heat map in ccRCC (Figure 7A and B). Promoter methylation levels of *G6PC* were significantly lower in primary ccRCC samples than normal samples (Figure 7C, *P *< 0.0001). Promoter methylation levels of *G6PC* significantly climbed with elevated individual cancer stage and were the highest in samples with stage 4 (Figure 7D). Promoter methylation levels of *G6PC* significantly climbed with elevated individual tumour grade and were the highest in samples with grade 4 (Figure 7E). Promoter methylation levels of *G6PC* were significantly higher in ccRCC samples with nodal metastasis compared with pN0 patients (Figure 7F, *P *< 0.05).

### 3.8 Potential role of G6PC in ccRCC immune microenvironment

**FIGURE 8:**
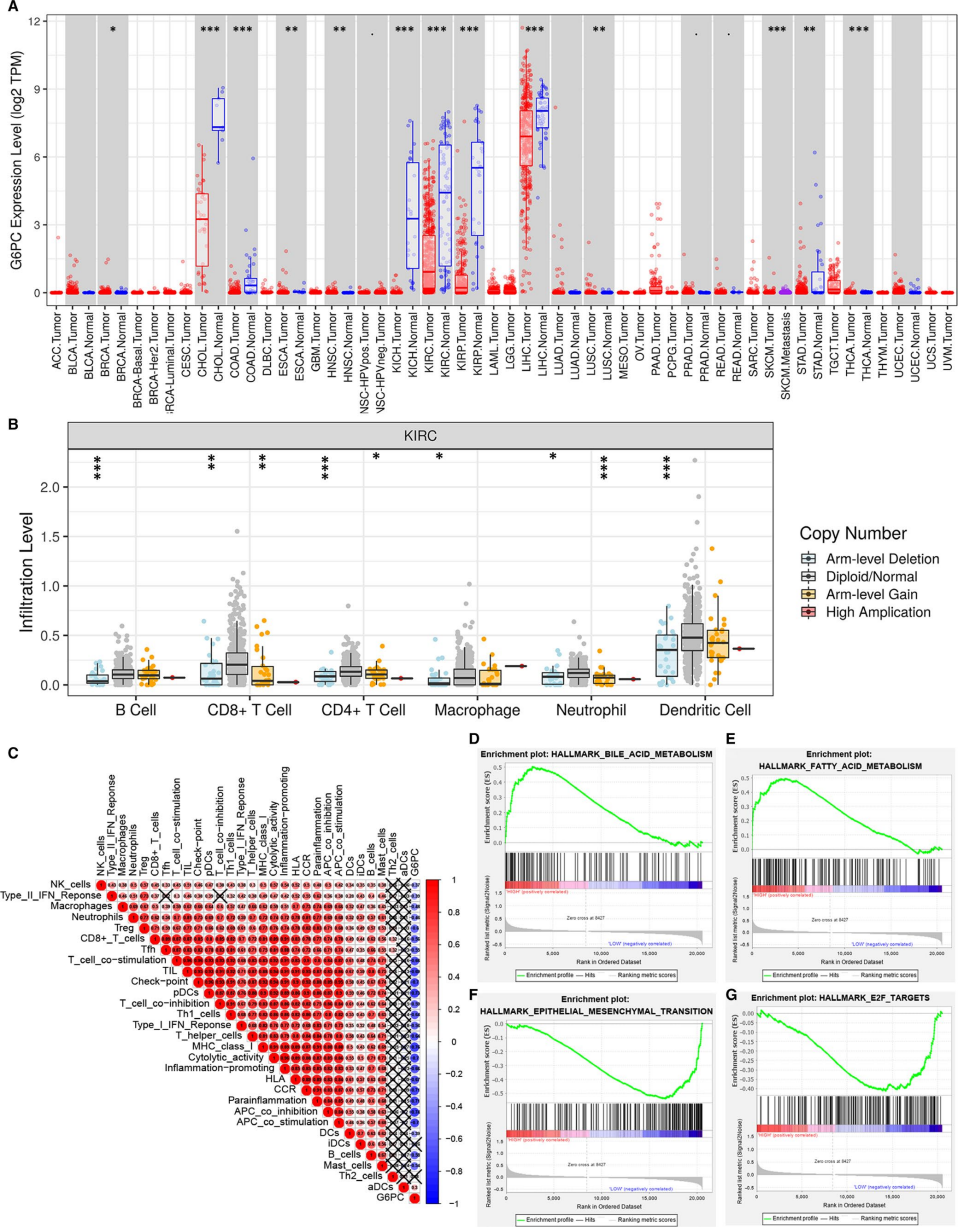
Potential role of *G6PC* in pan‐cancers and ccRCC microenvironment. A, As the main regulator of glucose production in the liver, high *G6PC* active expression is found in liver and kidney tissues. *G6PC* expression is significantly higher in normal tissue compared with renal cell carcinoma (KIRC, KIRP, KICH), while significantly lower in normal samples compared with hepatocellular carcinoma and cholangiocarcinoma. B, Copy number alteration of *G6PC* significantly correlated with environmental immune cells infiltration level. Elevated arm‐level deletion of *G6PC* leads to inferior B cell, CD8^+^ cells, CD4^+^ cells, macrophage, neutrophil, dendritic cells infiltration compared with normal samples (*P *< 0.05). C, *G6PC* significantly participates in abnormal immune infiltration of ccRCC cells and microenvironment, showing significantly negative association with check‐point immune signatures, dendritic cells, Th1 cells, MHC class I, cytolytic activity, inflammation promotion, HLA, APC co‐inhibition and co‐stimulation activities (cor.<−0.7). D‐G, GSEA indicated that *G6PC* significantly involved in several signal pathways, including bile acid metabolism, fatty acid metabolism, epithelial mesenchymal transition and E2F targets in ccRCC. ccRCC, clear cell renal cell carcinoma; KIRC, kidney renal clear cell carcinoma; KIRP, kidney renal papillary cell carcinoma; KICH, kidney Chromophobe; GSEA, gene set enrichment analysis

As the main regulator of glucose production, *G6PC* is highly expressed in liver and kidney tissues. *G6PC* expression is significantly higher in normal tissue compared with renal cell carcinomas, while significantly lower in normal samples compared with hepatocellular carcinoma and cholangiocarcinoma (Figure 8A). At the same time, copy number alteration of *G6PC* significantly correlated with environmental immune cells infiltration level (Figure 8B). Elevated arm‐level deletion of *G6PC* leads to inferior B cell, CD8^+^ cells, CD4^+^ cells, macrophage, neutrophil, dendritic cells infiltration compared with normal samples (*P *< 0.05). In addition, *G6PC* significantly participates in abnormal immune infiltration of ccRCC cells and microenvironment, showing significantly negative association with check‐point immune signatures, dendritic cells, Th1 cells, MHC class I, cytolytic activity, inflammation promotion, HLA, APC co‐inhibition and co‐stimulation activities (cor.< −0.7, Figure 8C). Moreover, GSEA indicated that *G6PC* significantly involved in several signal pathways, including bile acid metabolism, fatty acid metabolism, epithelial mesenchymal transition and E2F targets in ccRCC (Figure 8D‐G). A total of 100 up‐ and down‐regulated genes associated with differential *G6PC* expression were then visualized in ccRCC (Figure S3). Spearman's correlation and estimated statistical significance between *G6PC* expression and related genes and markers of immune cells were displayed in ccRCC patients using TIMER (**Table** **1**).

**TABLE 1:** Immune cells infiltrations in relationship to G6PC expression

Description	Gene markers	G6PC			
		None		Purity	
		Cor	*P*	Cor	*P*
CD8 + T cell	CD8A	−0.032	.468	−0.004	.94
	CD8B	−0.008	.862	0.022	.637
T cell (general)	CD3D	−0.081	.061	−0.052	.204
	CD3E	−0.067	.124	−0.037	.427
	CD2	−0.071	.104	−0.042	.369
B cell	CD19	−0.18	****	−0.151	**
	CD79A	−0.222	****	−0.209	****
Monocyte	CD86	−0.157	***	−0.142	**
	CD115 (CSF1R)	−0.146	***	−0.132	**
TAM	CCL2	0.083	.054	0.108	*
	CD68	−0.084	.054	−0.126	**
	IL10	−0.109	*	−0.087	.062
M1 Macrophage	INOS (NOS2)	0.194	****	0.216	****
	IRF5	−0.089	*	−0.112	*
	COX2 (PTGS2)	−0.252	****	−0.212	****
M2 Macrophage	CD163	−0.092	*	−0.095	*
	VSIG4	−0.232	****	−0.234	****
	MS4A4A	−0.136	**	−0.12	**
Neutrophils	CD66b (CEACAM8)	0.064	.138	0.047	.311
	CD11b (ITGAM)	−0.093	*	−0.085	.069
	CCR7	−0.085	*	−0.08	.087
Natural killer cell	KIR2DL1	0.108	*	0.089	.055
	KIR2DL3	0.087	*	0.065	.163
	KIR2DL4	−0.057	.192	−0.053	.252
	KIR3DL1	0.163	***	0.137	**
	KIR3DL2	0.094	*	0.077	.101
	KIR3DL3	−0.052	.232	−0.051	.276
	KIR2DS4	0.025	.567	0.02	.67
Dendritic cell	HLA‐DPB1	0.025	.572	0.038	.411
	HLA‐DQB1	0.073	.091	0.081	.082
	HLA‐DRA	0.022	.615	0.028	.553
	HLA‐DPA1	0.04	.355	0.062	.186
	BDCA‐1 (CD1C)	0.148	***	0.185	****
	BDCA‐4 (NRP1)	0.128	**	0.136	**
	CD11c (ITGAX)	−0.125	**	−0.123	**
Th1	T‐bet (TBX21)	0.076	.079	0.089	.057
	STAT4	−0.108	*	−0.108	*
	STAT1	−0.061	.163	−0.048	.304
	IFN‐γ (IFNG)	−0.089	*	−0.078	.095
	TNF‐α (TNF)	0.008	.861	0.023	.623
Th2	GATA3	−0.173	****	−0.083	.073
	STAT6	0.08	.066	0.056	.227
	STAT5A	−0.167	***	−0.151	**
	IL13	−0.009	.844	−0.027	.565
Tfh	BCL6	−0.198	****	−0.238	****
	IL21	−0.137	**	−0.13	**
Th17	STAT3	−0.091	*	−0.081	.081
	IL17A	−0.044	.31	−0.022	.636
Treg	FOXP3	−0.217	****	−0.212	****
	CCR8	−0.111	*	−0.106	*
	STAT5B	0.343	****	0.325	****
	TGFβ (TGFB1)	−0.325	****	−0.315	****
T cell exhaustion	PD‐1 (PDCD1)	−0.068	.116	−0.051	.273
	CTLA4	−0.115	**	−0.102	*
	LAG3	−0.115	**	−0.095	*
	TIM‐3 (HAVCR2)	0.173	****	0.157	***
	GZMB	−0.043	.319	−0.034	.46

* *P*< 0.05; ** *P*< 0.01; *** *P*< 0.001; **** *P*< 0.0001.

## 4 DISCUSSION

The control of energy metabolism in human is a complex and cautious process, and metabolic disorders may lead to the occurrence and development of a variety of diseases.^18^ For instance, abnormal lipid metabolism may reduce growth and impair fertility, while disorders in glucose metabolism can lead to diabetes and hypertension.^19^, ^20^ Importantly, the relationship between metabolic reprogramming and tumorigenesis has been paid more and more attention in recent years. Steven L. Gonias et al claimed that activation of lipid metabolism promotes tumour cell survival and tumour progression in pancreatic cancer.^21^ Some studies found that abnormal glucose metabolism plays an key role in tumorigenesis.^22^, ^23^ Metabolic changes promote the proliferation of tumours microenvironment and also help us better understand the alterations of characteristics phenotypes and immune microenvironment of cancers.^24^ For example, the activation of PI3K/AKT/mTOR and other carcinogenic pathways is related to the changes of bioenergy pathways such as glycolysis, fatty acid and glutamine metabolism, which provides a new target for tumour therapy.^25^, ^26^, ^27^

ccRCC is one of the most common types of renal cell carcinoma in the world, and it is associated with poor prognosis because of its high metastasis and recurrence rate.^1^, ^28^ Metabolic reprogramming in ccRCC is most often associated with mutations in VHL, which occur in about 90% of cases.^29^ In VHL mutant diseases, activation of metabolic pathways mediated by HIF leads to the activation of pathways contrary to the effects of hypoxia in normoxic environments.^30^ Previous studies found that ccRCC produces energy mainly through the accumulation of lactic acid,^31^, ^32^ which is also called Warburg effect or aerobic glycolysis. HIF‐1α, as the obvious driving force behind the Warburg effect in ccRCC, increases the expression of GLUT‐1, thus promoting intracellular glucose uptake.^33^, ^34^ Interestingly, complex components in tumour microenvironment could exhibit metabolic stress on immune cells infiltrations, which can lead to immunosuppressive and tumour immune evasion.^24^ The increased expression of GLUT‐1 in ccRCC is associated with a decrease in the number of infiltrated CD8^+^T cells, suggesting that glucose metabolism may suppress the immune system through another mechanism in renal cell carcinoma.^35^ Normally, elevated glycolysis increases tumours immunity, immune check‐point factors (PD‐L1) expression levels on tumour cells, and thus imposed a favourable immunotherapy response in cancers.^36^ Thus, the relationship between metabolic reprogramming and ccRCC microenvironment was worthy of further exploration.

G6PC (Glucose‐6‐Phosphatase Catalytic Subunit) is a protein coding gene, and it is closely associated with glycogen storage disease^37^ and hypoglycaemia.^38^ Gross, D. N et al claimed that G6PC was related to FoxO1 signalling pathway^39^ and G6PC plays a key role in Hexose transport.^40^ This study found that expression of G6PC in ccRCC is much lower than that of normal tissues in multiple cohorts including TCGA, CPTAC, ICGC and FUSCC cohorts. And survival analyses indicated that expression level of G6PC was positively correlated with patients’ outcome, suggesting that G6PC may have tumour suppressive properties in ccRCC. Studies have focused on exploring the biological significance of G6PC. Ting Guo et al ^41^ found that G6PC plays a dual role in both glucose metabolism and cell cycle regulation in ovarian cancer, which makes it a promising therapeutic target. Glycogen storage disease type I (GSDI) is a rare hereditary pathology characterized by glucose‐6‐phosphatase (G6Pase) deficiency. Monika Gjorgjieva et al found occurrence of ccRCC in mouse model with a kidney‐specific G6Pase deficiency (K. G6pc‐/‐ mice).^42^ It is not a unique instance, but has its counterpart. Cho Jun‐Ho *et al* ^43^ also claimed that G6PC could inhibit the occurrence of hepatic carcinoma, which is compatible with our hypothesis. In view of the possible inhibitory effect on tumour cells of G6PC, it may shed light on the management of ccRCC.

Thus, our research has some limitations. The main thing is the retrospective design of this study. Multicenter prospective studies are needed to verify the conclusions. Also, we did not validate prognostic specificity and sensitivity of MPMs in ccRCC patients from a real‐world cohort; thus, we presented prognostic value of MPMs in CRTAC and validate role of G6PC in > 1000 ccRCC patients from TCGA, CPTAC, RECA‐EU, HPA and FUSCC (validation cohort in China) cohorts. In addition, there is an urgent need for in vitro and in vivo experiments to explore potential effective functions of G6PC and reveal the underlying mechanisms.

## 5 CONCLUSION

In conclusion, this study first provided the opportunity to comprehensively elucidate the prognostic MDEGs landscape, established novel prognostic model MPMs using large‐scale ccRCC transcriptome data and identified G6PC as potential prognostic targets in 1040 ccRCC patients from multiply cohorts. These finding could assist in managing risk assessment and shed valuable insights into treatment strategies of ccRCC.

## CONFLICT OF INTERESTS

The authors declare no competing interests.

## AUTHORS’ CONTRIBUTIONS

The work was performed in co‐operation with all authors. YDW, ZHL and QYY defined research topics, discussed analysis, supervise studies, provided funding and revised manuscript. XWH, XY and TX drafted the manuscript, analysed data, interpreted and validated the results. AA, LWR and CDL assisted in performing data collection, statistical analysis and reference collection. WHK and SGH helped in IHC analysis and patients information collection from FUSCC. All authors read and approved the final manuscript. Wen‐Hao Xu: Data curation (equal); Formal analysis (equal); Investigation (equal); Methodology (equal); Resources (equal); Software (equal); Visualization (equal); Writing‐original draft (lead). Yue Xu: Data curation (equal); Formal analysis (equal); Investigation (equal); Methodology (equal); Resources (equal); Software (equal); Validation (equal); Visualization (equal). Xi Tian: Data curation (equal); Formal analysis (equal); Methodology (equal); Resources (equal); Software (equal); Validation (equal); Visualization (equal). Aihetaimujiang Anwaier: Data curation (supporting); Formal analysis (supporting); Investigation (equal); Methodology (supporting); Software (supporting); Writing‐original draft (supporting). Wangrui Liu: Data curation (supporting); Formal analysis (supporting); Methodology (supporting); Software (equal); Visualization (supporting). Jun Wang: Conceptualization (supporting); Investigation (supporting); Methodology (equal); Software (supporting); Visualization (supporting). Wen‐Kai Zhu: Conceptualization (supporting); Data curation (supporting); Investigation (supporting); Methodology (supporting); Project administration (supporting). Da‐Long Cao: Conceptualization (supporting); Investigation (supporting); Methodology (supporting); Resources (equal); Validation (supporting). Hong‐Kai Wang: Data curation (supporting); Investigation (supporting); Methodology (supporting); Resources (supporting); Supervision (supporting). Guo‐Hai Shi: Conceptualization (supporting); Methodology (supporting); Project administration (supporting); Resources (supporting); Supervision (supporting). Yuan‐Yuan Qu: Conceptualization (equal); Funding acquisition (equal); Project administration (equal); Resources (equal); Supervision (equal); Writing‐review & editing (equal). Hai‐Liang Zhang: Conceptualization (equal); Funding acquisition (equal); Project administration (equal); Resources (equal); Supervision (equal); Writing‐review & editing (equal). Ding‐Wei Ye: Conceptualization (equal); Project administration (equal); Resources (equal); Supervision (equal); Writing‐review & editing (equal).

## DECLARATIONS

Ethics approval and consent to participate: Study Ethics procedures were approved by Fudan University Shanghai Cancer Center (FUSCC, Shanghai, China). Written informed consents were acquired from online open‐access TCGA, CPTAC and RECA‐EU (available in ICGC database) included in this study.

## PATIENT CONSENT FOR PUBLICATION

Not applicable.

## Supporting information

Fig S1

Fig S2

Fig S3

Table S1

## Data Availability

The data sets analysed in this study were obtained from online open‐access databases or corresponding authors upon reasonable request.
