# Supplementary figures and images for: Large‐scale transcriptome profiles reveal robust 20‐signatures metabolic prediction models and novel role of G6PC in clear cell renal cell carcinoma
Source: J Cell Mol Med. 2020 Jun 21;24(16):9012–27. doi: 10.1111/jcmm.15536 (PMC7417710; doi:10.1111/jcmm.15536)

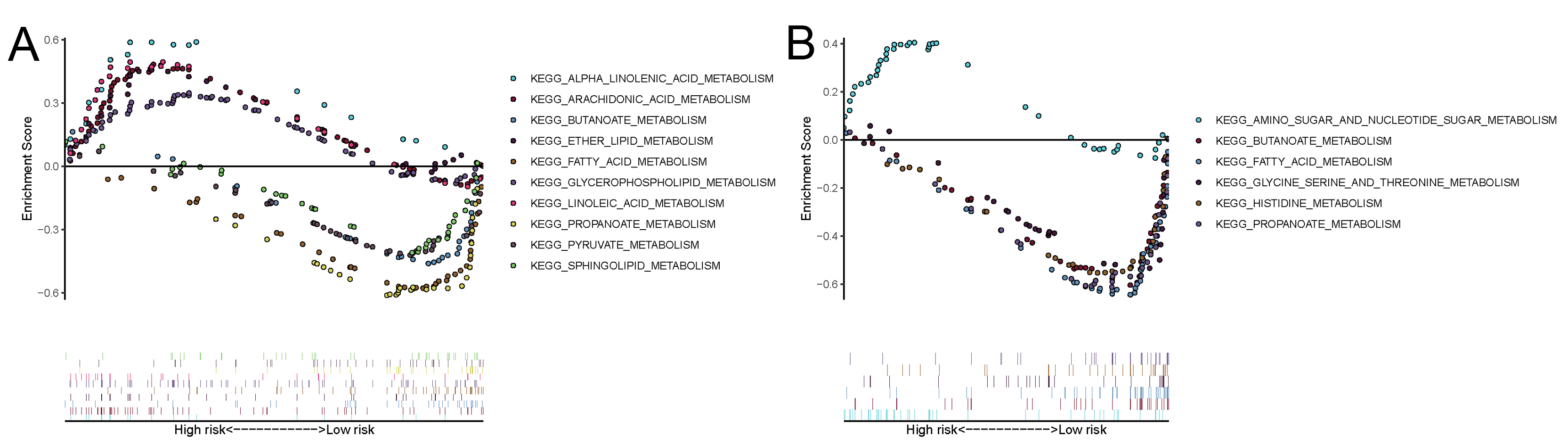

Supplement: Supplementary file 1 — Fig S1 [file JCMM-24-9012-s001.tif]

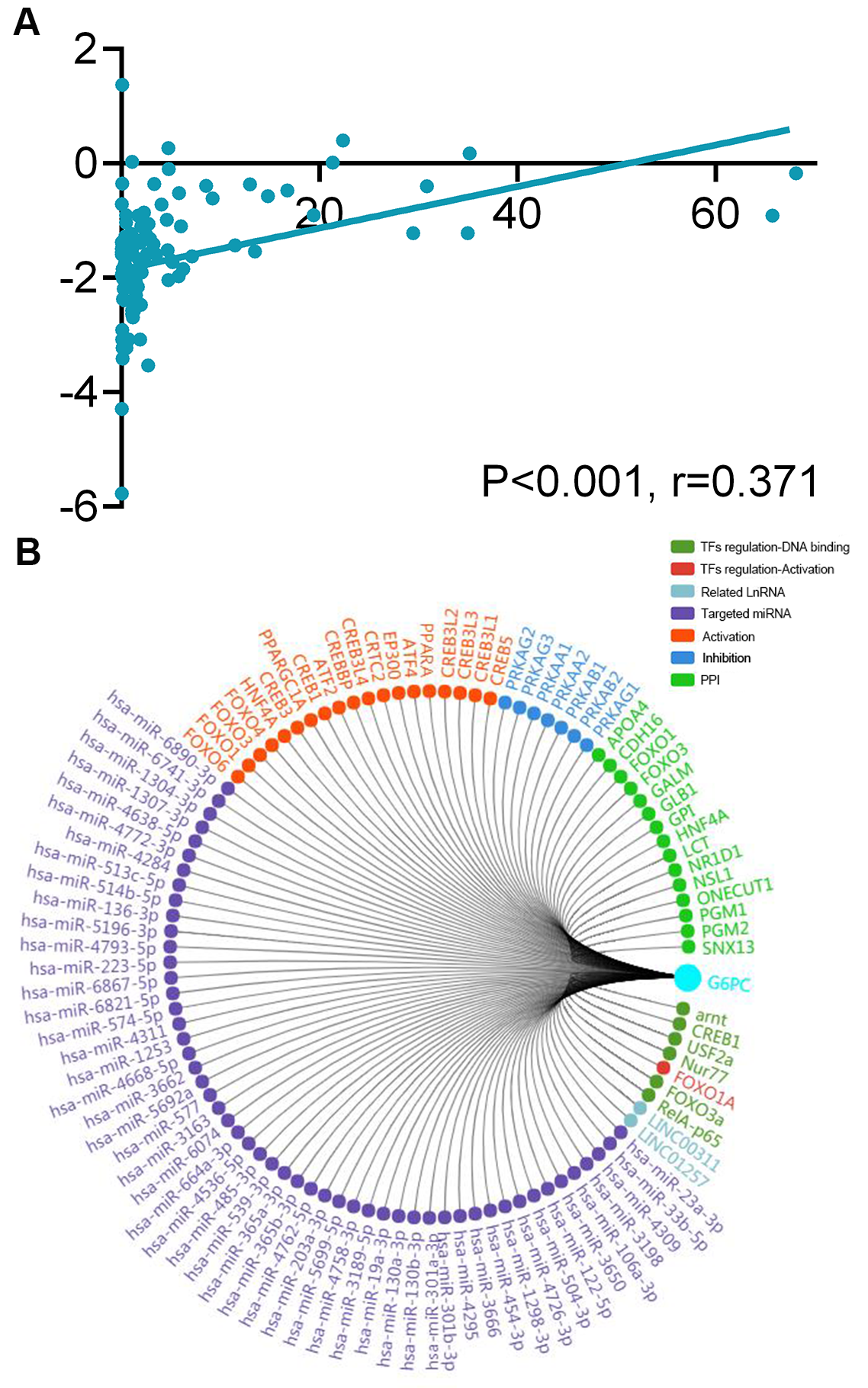

Supplement: Supplementary file 2 — Fig S2 [file JCMM-24-9012-s002.tif]

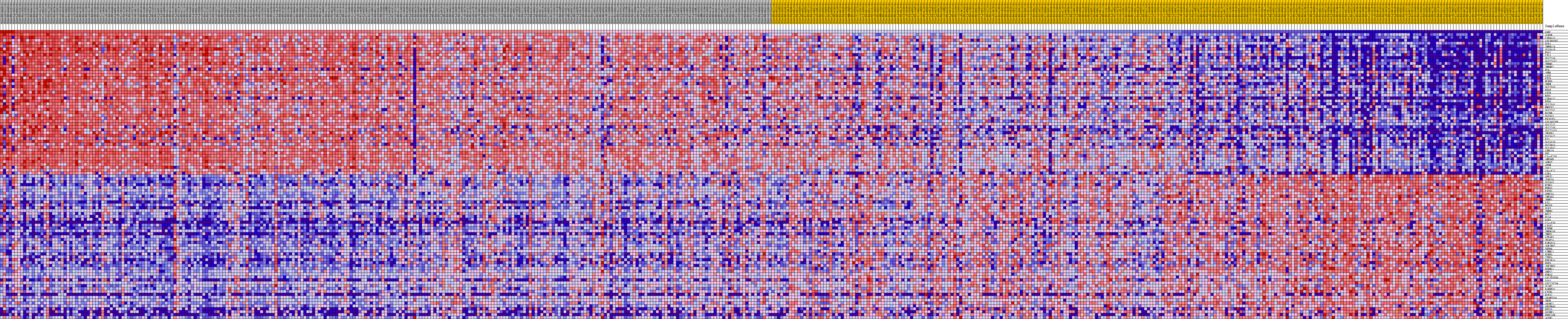

Supplement: Supplementary file 3 — Fig S3 [file JCMM-24-9012-s003.tif]
