# Supplementary material for: Large‐scale transcriptome profiles reveal robust 20‐signatures metabolic prediction models and novel role of G6PC in clear cell renal cell carcinoma
Source: J Cell Mol Med. 2020 Jun 21;24(16):9012–27. doi: 10.1111/jcmm.15536 (PMC7417710; doi:10.1111/jcmm.15536)
Supplement: Supplementary file 4 — Table S1 [file JCMM-24-9012-s004.docx]

**Table S1.** Clinicopathological characteristics in relation to G6Pase expression status in 322 ccRCC patients from FUSCC cohort.

| **Variable** | Entire group (n=320) | G6Pase expression | | χ^2^ | *P* value |
| --- | --- | --- | --- | --- | --- |
|  |  | Low expression (n=180) | High expression (n=142) |  |  |
| Age at surgery (y, median±SD) |  | 56.5±11.2 | 55.5±12.1 |  |  |
| Sex (n, %) |  |  |  | 0.700 | 0.403 |
| Male | 221 (68.6） | 127 (70.6) | 94 (66.2) |  |  |
| Female | 101 (31.4) | 53 (29.4) | 48 (33.8) |  |  |
| Laterality (n, %) |  |  |  | 0.528 | 0.467 |
| Left | 157 (48.8) | 91 (50.6) | 66 (46.5) |  |  |
| Right | 168 (51.2) | 89 (49.4) | 76 (53.5) |  |  |
| T stage at presentation (n, %) |  |  |  | **27.239** | **<0.001** |
| T1-T2 | 257 (79.8) | 125 (64.9) | 132 (93.0) |  |  |
| T3-T4 | 65 (20.2) | 55 (30.6) | 10 (7.0) |  |  |
| N stage at presentation (n, %) |  |  |  | **18.295** | **<0.001** |
| N0 | 279 (86.8) | 143 (79.4) | 136 (95.8) |  |  |
| N1 | 43 (13.4) | 37 (20.6) | 6 (4.2) |  |  |
| M stage at presentation (n, %) |  |  |  | **21.639** | **<0.001** |
| M0 | 260 (80.7) | 129 (71.7) | 131 (92.3) |  |  |
| M1 | 62 (19.3) | 51 (28.3) | 11 (7.7) |  |  |
| AJCC stage |  |  |  | **38.634** | **<0.001** |
| I-II | 246 (76.4) | 114 (63.3) | 132 (93.0) |  |  |
| III-IV | 76 (23.6) | 66 (36.7) | 10 (7.0) |  |  |
| ISUP grade (n, %) |  |  |  | **8.795** | **0.003** |
| 1-2 | 156 (48.4) | 74 (41.1) | 82 (57.7) |  |  |
| 3-4 | 166 (51.6) | 106 (58.9) | 60 (42.3) |  |  |

ccRCC, clear cell renal cell carcinoma; FUSCC, Fudan University Shanghai Cancer Center; AJCC, American Joint Committee on Cancer; *P* value less than 0.05 was considered as statistically significance, and marked in bold.
